# Supplementary material for: Genetic Adaptation of Coxsackievirus B1 during Persistent Infection in Pancreatic Cells
Source: Microorganisms. 2020 Nov 15;8(11):1790. doi: 10.3390/microorganisms8111790 (PMC7697981; doi:10.3390/microorganisms8111790)
Supplement: Supplementary file 1 [file microorganisms-08-01790-s001.pdf]

Supplementary material

2A

A2V	F3L	Y11C	Y11H	N14T	T46I	A47V	C50R	K70T	H71Y	G80R	E86G	Y89H	V120I	G130D	A147V
CVB1 10796, PANC-1	CVB1 10797, PANC-1	CVB1 ATCC E1, PANC-1	CVB1 ATCC, 1.1B4	CVB1 10802, PANC-1	CVB1 10796, PANC-1	CVB1 ATCC E2, PANC-1	CVB1 10796, PANC-1	CVB1 ATCC, 1.1B4	CVB1 ATCC E1, PANC-1	CVB1 10802, 1.1B4	CVB1 10796, PANC-1	CVB1 10796, PANC-1	CVB1 10802, 1.1B4	CVB1 10802, 1.1B4	CVB1 10796, 1.1B4
												CVB1 10796, 1.1B4			

2B

N11H	S15L	I50V	K58R	Y91H	D92G
CVB1 ATCC, 1.1B4	CVB1 ATCC E1, PANC-1	CVB1 10802, PANC-1	CVB1 10796, PANC-1	CVB1 ATCC, 1.1B4	CVB1 10796, PANC-1
	CVB1 ATCC E2, PANC-1				CVB1 10796, 1.1B4
	CVB1 ATCC, 1.1B4				

2C

L5F	T9A	V34F	P54S	S58N	S130G	L150F	M175L	V187I	L212M
CVB1 ATCC E1, PANC-1	CVB1 ATCC E2, PANC-1	CVB1 10796, 1.1B4	CVB1 10802, 1.1B4	CVB1 10796, 1.1B4	CVB1 ATCC E1, PANC-1	CVB1 ATCC E2, PANC-1	CVB1 10796, PANC-1	CVB1 ATCC, 1.1B4	CVB1 ATCC, 1.1B4
CVB1 ATCC, 1.1B4							CVB1 10796, 1.1B4		

P216S	I251V	M253L	K259R	T268A	V276A	R296G	M310L	Y314H
CVB1 10802, PANC-1	CVB1 ATCC, 1.1B4	CVB1 10802, 1.1B4	CVB1 10797, PANC-1	CVB1 ATCC E1, PANC-1	CVB1 10802, PANC-1	CVB1 10797, PANC-1	CVB1 10802, 1.1B4	CVB1 10802, PANC-1
								CVB1 10802, 1.1B4

Figure S1. Mutations on 2A, 2B and 2C non-structural proteins in all CVB1 PIDVs established in TP2. The location and the corresponding amino acid substitution are shown in the top line and the corresponding PIDVs are presented below. I50V in 2B, marked with grey color, is in hydrophobic domain of and K58R in hydrophilic domain. M175L, marked with grey color in 2C is in ATPase motif B region.

Virus	$TCID_{50}$/ml
ATCC E1-PIDV[PANC-1]	1.86E+05
ATCC E2-PIDV[PANC-1]	8.62E+05
10796-PIDV[PANC-1]	1.92E+06

Table S1. The 50% endpoint titers for CVB1 ATCC and CVB1 10796 viruses in PANC-1 cell model (in $TCID_{50}$/ml) measured by $TCID_{50}$ assay for 6.8 months post infection.

Virus	Cell type	CVB1 reads	Human reads[1]	mRNA	CVB/Human%[2]
CVB1 ATCC E1	PANC-1	18258	574736	844	3.20 %
CVB1 ATCC E2	PANC-1	15664	600530	1348	2.60 %
CVB1 10796	PANC-1	31963	547795	1232	5.80 %
CVB1 10802	PANC-1	85084	737236	846	11.50 %
CVB1 10797	PANC-1	76878	744692	697	10.30 %

CVB1 ATCC	1.1B4	118737	702899	1265	16.70 %
CVB1 10796	1.1B4	140833	661179	2086	21.20 %
CVB1 10802	1.1B4	220998	671472	2010	32.80 %

1. GRCH37, human reference genome. 2. CVB1/(GRCH37+mRNA)

Table S2. Human alignment is processed from Vipie virome pipeline [1] based on de novo Velvet [2] contig mapping using minimum Kmer 51 (data is available upon request). The mean CVB/Human Proportions of all samples are 13.01% (PANC-1 6.68% and 1.1B4 23.57%).

References

1. Lin, J.; Kramna, L.; Autio, R.; Hyoty, H.; Nykter, M.; Cinek, O. Vipie: web pipeline for parallel characterization of viral populations from multiple NGS samples. *BMC Genomics* **2017***, 18*, 378-7, DOI 10.1186/s12864-017-3721-7 [doi].

2. Zerbino, D.R.; Birney, E. Velvet: algorithms for de novo short read assembly using de Bruijn graphs. *Genome Res* **2008***, 18*, 821-829, DOI 10.1101/gr.074492.107 [doi].
